# Right Atrial Fluorodeoxyglucose Uptake Is a Risk Factor for Stroke and Improves Prediction of Stroke Above the CHA_2_DS_2_-VASc Score in Patients With Atrial Fibrillation

**DOI:** 10.3389/fcvm.2022.862000

**Published:** 2022-07-08

**Authors:** Bing Wang, Yiduo Xu, Peng Wan, Shan Shao, Feifei Zhang, Xiaoliang Shao, Jianfeng Wang, Yuetao Wang

**Affiliations:** ^1^Department of Nuclear Medicine, The Third Affiliated Hospital of Soochow University, Changzhou, China; ^2^Institute of Clinical Translation of Nuclear Medicine and Molecular Imaging, Soochow University, Changzhou, China; ^3^Department of Cardiology, The Third Affiliated Hospital of Soochow University, Changzhou, China

**Keywords:** stroke, atrial fibrillation, inflammation, ^18^F-fluorodeoxyglucose, positron emission tomography/computed tomography

## Abstract

**Background:**

Atrial fibrillation (AF) is a common arrhythmia, and its most severe and dreaded complication is stroke. The CHA_2_DS_2_-VASc score is currently recommended for stroke risk assessment in AF. We aimed to explore the relationship between atrial FDG uptake and stroke and whether atrial FDG uptake could provide incremental value above the CHA_2_DS_2_-VAS score to predict stroke in AF by ^18^F-fluorodeoxyglucose (^18^F-FDG) positron emission tomography/computed tomography (PET/CT).

**Materials and Methods:**

From September 2017 to December 2020, we retrospectively enrolled 230 patients (115 with AF and 115 without AF as the non-AF group, matched for the date of PET/CT examination and the basic characteristics of the patient) who underwent ^18^F-FDG PET/CT due to tumor screening or preoperative staging after prolonged fasting and followed up for at least 12 months from the date of PET/CT examination; the endpoint event is the occurrence of stroke. We visually and quantitatively analyzed ^18^F-FDG uptake in the right and left atria (RA/LA), right and left atrial appendage (RAA/LAA), right and left ventricle (RV/LV), and collected clinical features. In addition, according to the endpoint event (stroke), the enrolled population was divided into the stroke group and non-stroke group, and relevant clinical features and atrial FDG uptake indicators of the two groups were analyzed. Univariate and multivariate Cox regression analyzes were used to analyze the risk factors of stroke events. The Kaplan–Meier survival curve of atrial FDG uptake was drawn, and the log-rank method was used to compare the differences in the survival curves of the two groups. Receiver operating characteristic (ROC) curves were used to examine the discriminatory power of atrial FDG uptake in predicting stroke and determine whether the addition of atrial FDG uptake improves predictive value beyond the CHA_2_DS_2_-VASc score for stroke.

**Results:**

In the AF group, more than half of patients had RA FDG uptake and one-fifth had LA FDG uptake, while one patient had RA FDG uptake and two patients had LA FDG uptake in the non-AF group. In quantitative analysis, the maximum standardized uptake value (SUV_*max*_) of the RA and LA in the AF group was significantly higher than that of the non-AF group (all *P* < 0.001). We followed up the patients for 28 ± 10 months, and finally, 31 patients had stroke. In the stroke group, atrial fibrillation, RA SUV_*max*_, RAA SUV_*max*_, LAA SUV_*max*_, age ≥ 75 years, and left atrial dilation were significantly higher than those of the non-stroke group (all *P* < 0.05). Multivariate Cox regression analysis showed that high RA SUV_*max*_ (RA SUV_*max*_ ≥ 2.62) was an independent risk factor for stroke (HR = 4.264, 95% CI 1.368–13.293, *P* = 0.012). By using the log-rank test, patients with high RA SUV_*max*_ had a significantly higher incidence of stroke compared with patients with low RA SUV_*max*_ (*P* < 0.001). Addition of high RA SUV_*max*_ to the CHA_2_DS_2_-VASc score could predict stroke more effectively, with a larger AUC 0.790 (*P* < 0.001).

**Conclusion:**

This study found a significant correlation between atrial FDG uptake and AF, especially in RA. Meanwhile, RA FDG uptake is an independent risk factor for stroke, and patients with high RA SUV_*max*_ have a significantly higher risk of stroke. Moreover, RA FDG uptake improves prediction of stroke above the CHA_2_DS_2_-VASc score in patients with AF.

## Introduction

Atrial fibrillation (AF) is the most common cardiac arrhythmia, with a global incidence of approximately 2% (1). With increased average global life expectancy and longer survival with chronic conditions, the incidence and prevalence of AF also increase, which has brought a huge burden to the global economy and health (2). AF not only causes heart failure and myocardial ischemia but also induces the formation of atrial thrombus and systemic embolism, especially stroke. Stroke is the complication of AF, and 70–80% of patients with AF-related stroke suffer disability and even death (3). Therefore, it is critical for the prevention and treatment of stroke.

According to current guidelines, it is recommended to use the CHA_2_DS_2_-VASc [congestive heart failure, hypertension, age ≥ 75 years (double weight), diabetes, stroke (double weight), vascular disease, age 65–74 years, sex category (female)] score to assess the risk of stroke in patients with AF, which is based on the calculation of the score of the common risk factors (4). However, some studies show that the prediction effect is poor, and its score underestimates the true risk of stroke in patients with low scores (5, 6). The CHA_2_DS_2_-VASc score includes neither any imaging or biomarker parameters nor some of the less common clinical stroke risk factors. Studies have shown that indicators such as brain natriuretic peptide (BNP), atrial morphology, spontaneous echo contrast (SEC), and coronary artery calcium score (CACS) may further assist decision on anticoagulant therapy for patients with AF to prevent stroke (7, 8).

Recently, several studies have shown that inflammation predicts the development of AF and subsequent thromboembolism (9, 10). Inflammation plays an important role in the pathogenesis and progression of atherosclerosis, plaque rupture, platelet aggregation, and intravascular thrombosis, all of which increase the risk of stroke (11). Histological study has demonstrated that the expression of tissue factor induced by local inflammation is involved in the formation of thrombus in patients with AF (12). C-reactive protein (CRP), interleukin-6 (IL-6), and lipoprotein-associated phospholipase A2 (Lp-PLA2) are some of the inflammatory markers associated with stroke (13–15). A pathological biopsy is invasive, and blood inflammation indicators cannot accurately reflect inflammation in local tissues of the heart; therefore, non-invasive imaging techniques to detect local inflammation are essential.

Increasing evidence shows that ^18^F-fluorodeoxyglucose (^18^F-FDG) positron emission tomography/computed tomography (PET/CT) has important value in the diagnosis of inflammatory diseases, the judgment of inflammatory active and inactive periods, and the evaluation of therapeutic effects (16–18). Studies have shown that the degree of ^18^F-FDG uptake was linearly related to the density of inflammatory cells (19), so the maximum standardized uptake value (SUV_*max*_) of ^18^F-FDG can quantitatively reflect the inflammatory activity of tissues. Sinigaglia M et al. have analyzed the relationship between atrial FDG uptake and stroke in patients with AF by ^18^F-FDG PET/CT and found that increased atrial FDG uptake was associated with increased prevalence of stroke (20). However, the enrolled population was to assess cardiac inflammation or infection, and it was finally confirmed that the study population included valvular heart disease, coronary artery disease, cardiac sarcoidosis, and infective endocarditis. Those diseases and the associated confounders may have influenced atrial uptake. Moreover, this study lacked specific timing information, which limited the ability to assess the effect of time on the occurrence of events. It also did not compare the CHA_2_DS_2_-VASc score to assess the efficacy of RA FDG uptake in predicting stroke.

The purpose of this study is to evaluate the relationship between atrial FDG uptake and stroke in patients with atrial fibrillation, excluding cardiac and inflammatory diseases, and to detect whether atrial FDG uptake could provide incremental value above the CHA_2_DS_2_-VASc score to predict stroke.

## Materials and Methods

### Study Population

**FIGURE 1:**
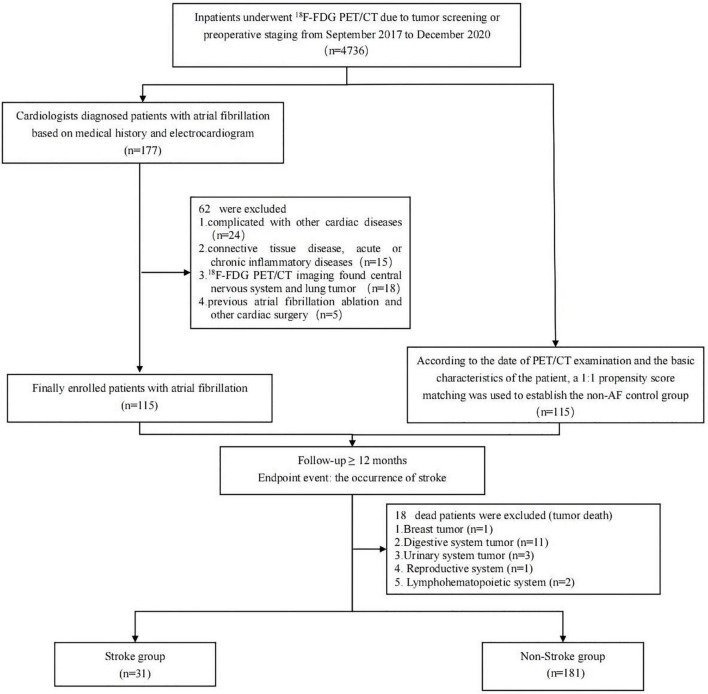
Study population.

From September 2017 to December 2020, we retrospectively enrolled 115 patients with AF who underwent ^18^F-FDG PET/CT in the Nuclear Medicine Department of the Third Affiliated Hospital of Soochow University due to tumor screening or preoperative staging (Figure 1). Inclusion criteria are as follows: (1) patients diagnosed with AF by a cardiologist based on their medical history and electrocardiogram, paroxysmal AF that terminates spontaneously or with intervention within 7 days of onset, and persistent AF that is continuously sustained beyond 7 days, including episodes terminated by cardioversion (drugs or electrical cardioversion) after ≥ 7 days(4); (2) electrocardiogram, echocardiography, and related hematological examinations within one week; (3) relevant clinical data; and (4) follow-up ≥ 12 months. Exclusion criteria are as follows: (1) age ≤ 18 years; (2) complicated with other cardiac diseases (such as coronary artery disease, congenital heart disease, valvular disease, and pericardial disease); (3) connective tissue disease, acute or chronic inflammatory diseases; (4) ^18^F-FDG PET/CT imaging found central nervous system tumor and lung tumor; (5) previous atrial fibrillation ablation and other cardiac surgery; and (6) death due to tumor during the follow-up. According to the date of PET/CT examination and the basic characteristics of the patient [gender, age, body mass index (BMI), heart rate, malignant tumor history, and fasting blood-glucose], a 1:1 propensity score matching (PSM) was used to establish the non-AF group. This retrospective study was approved by the Institutional Ethics Committee of the Third Affiliated Hospital of Soochow University [(2020) KD 034]. Informed consents were waived owing to the study’s retrospective nature.

### Clinical Data Collection

The clinical data of patients were collected through an electronic medical record system, including gender, age, height, weight, heart rate, fasting blood glucose, active smoking, active drinking, type of atrial fibrillation, heart failure, tumor [tumor type (head and neck, breast, digestive system, urinary system, reproductive system, and lymphohematopoietic system) and treatment for tumor (surgery, chemotherapy, and radiation therapy)], medical history (hypertension, diabetes, and dyslipidemia), and medication (antiplatelet, anticoagulant, hypolipidemic, and antihypertensive drugs).

### ^18^F-Fluorodeoxyglucose Positron Emission Tomography/Computed Tomography (^18^F-FDG PET/CT) Imaging

The German Siemens PET/CT device (Biograph mCT 64, 52-ring LSO crystal/64-slice spiral CT) was used for imaging. Patients fasted for more than 12 h before the examination and were injected ^18^F-FDG intravenously at a dose of 3.7 MBq/kg. After resting for 60 min in a quiet and comfortable environment, the patients maintained a supine position and breathed steadily. Imaging started with the 64-slice spiral CT (120 kV, 35 mA) for attenuation correction in all patients. After the CT scan, PET three-dimensional (3D) collection was performed, collecting 5–6 beds, 2.5 min/bed. The scanning range was from the top of the skull to the middle and upper part of the femur.

### ^18^F-Fluorodeoxyglucose Positron Emission Tomography/Computed Tomography (^18^F-FDG PET/CT) Imaging Analysis

The visual and quantitative analyzes of the image were performed using post-processing workstations (TureD software) by two PET/CT physicians blind to patients’ medical records.

In visual analysis (21), using a four-point grading system: grade 0, atrial ^18^F-FDG uptake was lower than the adjacent blood pool (background); grade 1, atrial ^18^F-FDG uptake was similar to the background; grade 2, atrial ^18^F-FDG uptake was slightly higher than the background; and grade 3, atrial ^18^F-FDG uptake was evidently higher than the background. Grade 2–3 atrial ^18^F-FDG uptake was defined as positive uptake. Ventricular positive uptake interpretation criteria are as aforementioned.

In quantitative analysis (22), the maximum standardized uptake value (SUV_*max*_) out of all slices was selected to represent the activity of the atrial. If no positive uptake could be found, three circular regions of interest (ROIs) with a diameter of 5 mm were placed on the wall of the atria. The measurement of the atrial appendage and ventricle was carried out as described previously. To obtain the background value of ^18^F-FDG uptake, a circular ROI with a diameter of 5 mm was placed on the ascending aorta cavity, and the mean SUV (SUV_*mean*_) was recorded (21). Thereafter, a target-to-background ratio (TBR) was calculated for the bilateral atria and atrial appendage (AA), respectively. The final value of the aforementioned measurement index was the average value measured by two physicians.

### Echocardiogram

Within one week of PET/CT examination, all selected patients completed echocardiography. The left ventricular ejection fraction (LVEF) and left atrial diameter (LAD) were calculated based on biplane two-dimensional echocardiography (Simpson’s method) (23).

### Follow-Up

All patients were followed up for at least 12 months from the date of PET/CT examination and were followed up by the outpatient clinic, readmission, or telephone. The endpoint event was the occurrence of stroke. Stroke includes transient ischemic attack (TIA) and thromboembolism, which are confirmed by clinicians through the patients’ symptoms and imaging examination (computed tomography or magnetic resonance imaging) (4).

### Statistical Analysis

SPSS Statistics (version 25; IBM) and MedCalc (version 20.015) were used to perform the statistical analysis. The Kolmogorov–Smirnoff test was used to assess the normality of the distribution of continuous variables. Continuous variables were presented as mean ± standard deviation (SD) or median (25th–75th percentile), and compared using Student’s *t*-test or the Mann–Whitney U test. Categorical variables were presented as percentages and compared using the chi-square test or Fisher’s exact test. The optimal cutoff value for cardiac FDG uptake was obtained by the Youden index of the receiver operating characteristic (ROC) curve and transformed into a categorical variable (Supplementary Table 1). The cardiac SUV_*max*_ ≥ optimal cutoff value was defined as high cardiac SUV_*max*_. Intra- and inter-observer reproducibility of FDG uptake measurement were assessed using the intraclass correlation coefficient (ICC).

Cox proportional hazards models were used to explore the relationship between baseline risk factors and stroke incidence. The significance of each risk factor was evaluated by univariate Cox regression analysis to investigate the independent variables affecting the occurrence of stroke events. Multivariate Cox proportional hazards regression analysis was used to further evaluate risk factors associated with stroke at the significant level (*P* < 0.1). The Kaplan–Meier survival curve was drawn to observe the survival curves of the two groups of RA SUV_*max*_ (high and low), and the log-rank method was used to compare the differences in the survival curves of the two groups. ROC curves were plotted to examine the discriminatory power of atrial FDG uptake in predicting stroke and determine whether the addition of atrial FDG uptake improves predictive value beyond the CHA_2_DS_2_-VASc score for stroke. A *p*-value < 0.05 was considered statistically significant.

## Results

### Clinical Characteristics of Study Population

The demographic, clinical, and ultrasound parameters of the study population are given in Table 1. Group 1 consisted of 115 patients with a history of AF (32.2% female, 69 ± 9 years, 70.4% with persistent AF, 29.6% with paroxysmal AF). Group 2 consisted of 115 patients without AF matched for the date of PET/CT examination and basic characteristics (34.8% female, 67 ± 7 years). Age, gender, BMI, heart rate, fasting blood glucose, active smoking, active drinking, hypertension, diabetes, dyslipidemia, serum cholesterol, and blood inflammation indicators (CRP, leukocytes, neutrophils, and lymphocytes) were not statistically different between the AF group and non-AF group (all *P* > 0.05). Moreover, there was no statistically significant difference in tumor type, treatment for tumor, and medication between the two groups (all *P* > 0.05) (Supplementary Table 2).

**TABLE 1:** Characteristics of study patients.

	Total population (*N* = 230)	AF group (*N* = 115)	Non-AF group (*N* = 115)	*P*-value
Age, years	68 (8)	69 (9)	67 (7)	0.125
Female, n (%)	77 (33.5)	37 (32.2)	40 (34.8)	0.675
BMI, kg/m^2^	22.88 (2.91)	22.92 (3.24)	22.84 (2.56)	0.837
Heart rate, bpm	76.23 (12.20)	76.25 (13.67)	76.21 (10.60)	0.979
Fasting blood-glucose, mmol/L	5.70(5.22−6.40)	5.70(5.23−6.30)	5.70(5.10−6.40)	0.747
Tumor, n (%)	102 (44.3)	48 (41.7)	54 (47.0)	0.426
Hypertension, n (%)	115 (50.0)	59 (51.3)	56 (48.7)	0.692
Diabetes, n (%)	37 (16.1)	17 (14.8)	20 (17.4)	0.590
Dyslipidemia, n (%)	46 (20.0)	23 (20.0)	23 (20.0)	1.000
Active smoking, n (%)	79 (34.3)	38 (33.0)	41 (35.7)	0.677
Active drinking, n (%)	40 (20.4)	23 (20.0)	24 (21.0)	0.870
Heart failure, n (%)	20 (8.7)	16 (13.9)	4 (3.4)	**0.010**
Left atrial dilation, n (%)	93 (40.4)	83 (72.2)	10 (8.7)	**0.000**
Triglyceride, mmol/L	1.21(1.02−1.46)	1.17(0.93−1.49)	1.21(1.04−1.36)	0.633
Total cholesterol, mmol/L	4.27 (0.63)	4.21 (0.77)	4.31 (0.44)	0.247
HDL cholesterol, mmol/L	1.46 (0.48)	1.52 (0.59)	1.41 (0.34)	0.097
LDL cholesterol, mmol/L	2.20 (0.67)	2.14 (0.81)	2.27 (0.48)	0.158
CRP, mg/L	4.30(3.50−5.50)	4.00(3.50−5.00)	4.60(3.50−5.90)	0.242
Leukocytes, 10^9^/L	5.89 (1.59)	5.88 (1.56)	5.91 (1.63)	0.882
Neutrophils, 10^9^/L	3.67(2.86−4.55)	3.62(2.86−4.59)	3.70(2.92−4.55)	0.574
Lymphocytes, 10^9^/L	1.49 (0.56)	1.51 (0.60)	1.47 (0.53)	0.250
LAD, mm	39.29 (7.17)	43.71 (6.82)	34.87 (4.15)	**0.000**
LVEF,%	63(60−65)	61(58−63)	64(62−66)	**0.000**

*AF, atrial fibrillation; BMI, body mass index; LA, left atria; HDL, high-density lipoprotein; LDL, low-density lipoprotein; CRP, C-reactive protein; LAD, left atrial diameter; LVEF, left ventricular ejection fraction.*

*P < 0.05.*

Compared with non-AF group, AF group had more patients with heart failure and left atrial dilation, and the differences were statistically significant (all *P* < 0.05). In the ultrasound parameters, the LAD of the AF group was higher than that of the non-AF group, while the LVEF was lower than that of the non-AF group, and the differences were statistically significant (all *P* < 0.05).

### Atrial ^18^F-Fluorodeoxyglucose (^18^F-FDG) Uptake and Atrial Fibrillation (AF)

For all enrolled patients, atrial visual analysis and quantitative analysis (the SUV_*max*_ and TBR values of the atria, AA, and ventricle) are listed in Table 2. Of the 115 patients with AF, 70 (60.9%) showed positive uptake in RA, 26 (22.6%) showed positive uptake in LA, and 24 (20.9%) showed positive uptake in both atria, whereas 34 (29.6%) showed no positive uptake in both atria. In the non-AF group, one patient showed positive uptake in the RA and two patients showed positive uptake in LA. The positive uptake rates of the RV in the AF group and the non-AF group were 2.6 and 4.3%, and the positive uptake rates of the LV were 50.4 and 40.9%, and there was no significant difference between the two groups (*P* > 0.05).

**TABLE 2:** Comparison of atrial ^18^F-fluorodeoxyglucose (^18^F-FDG) uptake in atrial fibrillation (AF) and non-AF groups.

	Total population (*N* = 230)	AF group (*N* = 115)	Non-AF group (*N* = 115)	*P*-value
**Visual analysis**				
RA positive uptake	71 (30.9)	70 (60.9)	1 (0.9)	**0.000**
RV positive uptake	8 (3.5)	3 (2.6)	5 (4.3)	0.719
LA positive uptake	28 (12.2)	26 (22.6)	2 (1.7)	**0.000**
LV positive uptake	105 (45.7)	58 (50.4)	47 (40.9)	0.186
**Quantitative analysis**				
RA SUV_*max*_	2.50 (1.82–3.59)	3.58 (2.70–4.80)	1.95 (1.63–2.20)	**0.000**
RAA SUV_*max*_	2.30 (1.90–3.03)	3.00 (2.36–4.22)	2.04 (1.70–2.30)	**0.000**
RV SUV_*max*_	2.48 (2.15–2.80)	2.50 (2.10–1.96)	2.47 (2.19–2.67)	0.726
LA SUV_*max*_	2.22 (1.78–2.75)	2.60 (2.00–3.30)	2.00 (1.71–2.48)	**0.000**
LAA SUV_*max*_	2.22 (1.90–2.76)	2.65 (2.20–3.30)	1.95 (1.74–2.23)	**0.000**
LV SUV_*max*_	4.97 (2.91–10.03)	5.70 (3.20–9.66)	3.94 (2.54–10.36)	0.052
TBR RA	1.29 (0.75–1.96)	1.82 (1.37–2.45)	0.75 (0.71–0.97)	**0.000**
TBR RAA	1.13 (0.83–1.82)	1.67 (1.17–2.14)	0.84 (0.74–0.97)	**0.000**
TBR RV	1.22 (0.99–1.41)	1.26 (1.07–1.35)	1.12 (0.95–1.35)	**0.004**
TBR LA	1.08 (0.80–1.51)	1.35 (1.09–1.86)	0.81 (0.73–1.07)	**0.000**
TBR LAA	1.05 (0.77–1.55)	1.39 (1.07–1.93)	0.81 (0.67–1.00)	**0.000**
TBR LV	2.18 (1.29–5.13)	3.09 (1.65–5.28)	1.86 (1.05–4.47)	**0.001**

*AF, atrial fibrillation; RA, right atrial; RV, right ventricle; LA, left atrial; LV, left ventricle; RAA, right atrial appendage; LAA, left atrial appendage; SUV_max_, maximum standardized uptake value; TBR, target-to-background ratio.*

*P < 0.05.*

**FIGURE 2:**
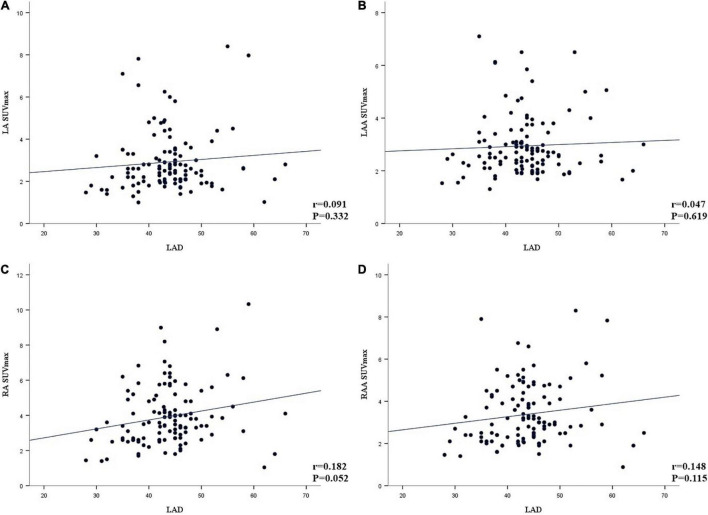
**(A–D)** Linear correlation analysis between the left atrial diameter (LAD) and the maximum standardized uptake value (SUV_*max*_) of left atria (LA), left atrial appendage (LAA), right atrial (RA), and right atria appendage (RAA) (A, LA; B, LAA; C, RA; D, RAA).

In agreement with the visual analysis, the SUV_*max*_ and TBR values of RA, RAA, LA, and LAA in the AF group were significantly higher than those of the non-AF group (all *P* < 0.001). The AF group has more patients with left atrial dilation than the non-AF group (72.2 vs. 8.7%, respectively, *P* < 0.001), but LAD was not correlated with LA SUV_*max*_, LAA SUV_*max*_, RA SUV_*max*_, and RAA SUV_*max*_ (Figure 2). There was no significant difference in RV SUV_*max*_ and LV SUV_*max*_ between the two groups (*P* > 0.05). However, compared with the non-AF group, the TBR (RV and LV) of patients in the AF group was higher, and the difference was statistically significant (*P* < 0.05).

### Atrial ^18^F-Fluorodeoxyglucose (^18^F-FDG) Uptake and Stroke

We followed up the patients for 28 ± 10 months from the date of PET/CT examination, during which 18 patients died (due to tumor) and 31 patients had stroke finally. After excluding dead patients, the enrolled patients were divided into two groups according to stroke (Table 3). The stroke group consisted of 31 patients with stroke during the follow-up (32.3% female, 71 ± 8 years, 77.4% with AF). Non-stroke consisted of 181 patients without stroke during the follow-up (33.7% female, 68 ± 9 years, 45.9% with AF). The incidence of stroke was more common in the AF group than the non-AF group (24 patients vs. 7 patients, respectively).

**TABLE 3:** Comparison of atria ^18^F-fluorodeoxyglucose (^18^F-FDG) uptake and stroke risk factors in stroke group and non-stroke group.

	Stroke group (*N* = 31)	Non-stroke group (*N* = 181)	*P*-value
Atrial fibrillation, n (%)	24 (77.4)	83 (45.9)	**0.001**
Tumor, n (%)	12 (38.7)	72 (39.8)	0.910
FDG uptake			
RA SUV_*max*_	3.60 (2.80-5.10)	2.30 (1.80-3.30)	**0.000**
RAA SUV_*max*_	2.84 (2.21-4.30)	2.30 (1.90-2.90)	**0.002**
RV SUV_*max*_	2.40 (1.90-2.88)	2.48 (1.29-2.77)	0.397
LA SUV_*max*_	2.35 (1.80-3.25)	2.26 (1.80-2.70)	0.676
LAA SUV_*max*_	2.55 (2.20-3.12)	2.20 (1.89-2.70)	**0.007**
LV SUV_*max*_	6.44 (3.05-11.25)	4.99 (2.83-10.09)	0.900
Stroke risk factors			
Age, n (%)			
74>Age ≥ 65 years	12 (38.7)	93 (51.4)	0.192
≥ 75 years	11 (35.5)	33 (18.2)	**0.029**
Sex (female), n (%)	10 (32.3)	61 (33.7)	0.875
Active smoking, n (%)	12 (38.7)	60 (33.1)	0.546
Active drinking, n (%)	6 (19.4)	40 (22.1)	0.732
Hypertension, n (%)	18 (58.0)	90 (49.7)	0.391
Diabetes, n (%)	6 (19.4)	29 (16.0)	0.644
Dyslipidemia, n (%)	4 (12.9)	38 (21.0)	0.296
Left atrial dilation, n (%)	18 (58.1)	67 (37.0)	**0.000**
Heart failure, n (%)	4 (12.9)	11 (6.1)	0.171

*RA, right atrial; RAA, right atrial appendage; RV, right ventricle; LA, left atrial; LAA, left atrial appendage; LV, left ventricle; SUV_max_, maximum standardized uptake value; TBR, target-to-background ratio.*

*P < 0.05.*

In the stroke group, atrial fibrillation, RA SUV_*max*_, RAA SUV_*max*_, LAA SUV_*max*_, age ≥ 75 years, and left atrial dilation were significantly higher than those of the non-stroke group (all *P* < 0.05). In visual analysis, only RA positive uptake was statistically different between the two groups (*P* < 0.001) (Supplementary Table 3). In visual analysis or quantitative analysis, there was no statistically significant difference in ventricular uptake (all *P* > 0.05). Moreover, tumor type, treatment for tumor, and medication were not statistically different between the stroke group and the non-stroke group (all *P* > 0.05) (Supplementary Table 4).

### Multivariate Analysis Predicts the Occurrence of Stroke

**FIGURE 3:**
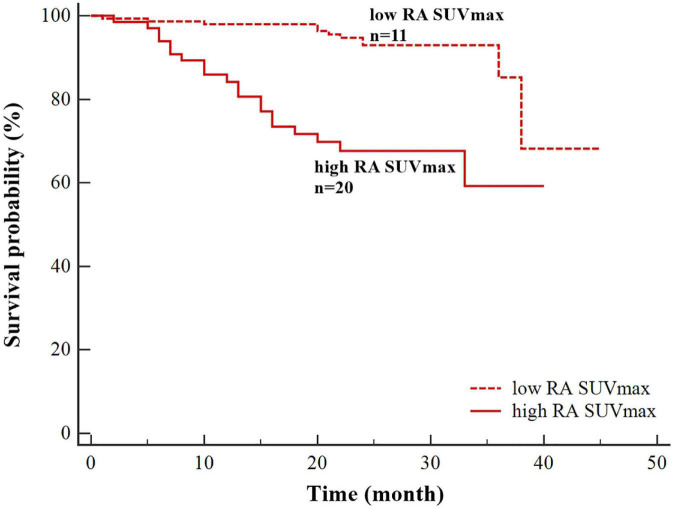
Kaplan–Meier survival curves for stroke based on right atrial maximum standardized uptake value (RA SUV_*max*_) (high RA SUV_*max*_ ≥ 2.62;low RA SUV_*max*_ < 2.62).

Univariate Cox regression analyzes demonstrated that atrial fibrillation (HR = 4.433, *P* = 0.001), high RA SUV_*max*_ (HR = 5.213, *P* = 0.000), high RAA SUV_*max*_ (HR = 5.330, *P* = 0.006), high LAA SUV_*max*_ (HR = 2.665, *P* = 0.012), age ≥ 75 years (HR = 2.488, *P* = 0.007), left atrial dilation (HR = 2.570, *P* = 0.010), and heart failure (HR = 2.986, *P* = 0.044) were significantly associated with the occurrence of stroke (Table 4). Multivariate Cox regression analysis showed that high RA SUV_*max*_ was an independent risk factor for stroke (HR = 4.264, 95% CI 1.368–13.293, *P* = 0.012). By using the log-rank test, patients with high RA SUV_*max*_ (RA SUV_*max*_ ≥ 2.62) (median time of stroke = 30.39 months) had a significantly higher rate of stroke than patients with low RA SUV_*max*_ (RA SUV_*max*_ < 2.62) (median time of stroke = 41.14 months) (*P* < 0.001, Figure 3).

**TABLE 4:** Univariate and multivariate Cox analyzes of prognostic risk factors for stroke.

	Univariate analysis		Multivariate analysis	
	HR (95% CI)	*P* value	HR (95% CI)	*P*-value
Atrial fibrillation, n (%)	4.433 (1.897–10.360)	**0.001**	1.672 (0.482–5.800)	0.418
Tumor, n (%)	1.128 (0.557–2.186)	0.738		
FDG uptake				
High RA SUV_*max*_	5.213 (2.488–10.921)	**0.000**	4.264 (1.368–13.293)	**0.012**
High RAA SUV_*max*_	5.330 (1.618–17.560)	**0.006**	0.870 (0.607–1.247)	0.448
High RV SUV_*max*_	1.361 (0.650–2.851)	0.414		
High LA SUV_*max*_	1.547 (0.747–3.204)	0.240		
High LAA SUV_*max*_	2.665 (1.245–5.706)	**0.012**	1.262 (0.487–3.270)	0.632
High LV SUV_*max*_	1.678 (0.829–3.399)	0.150		
Stroke risk factors				
Age, n (%)				
74>Age ≥ 65 years	0.541 (0.262–1.118)	0.097	0.826 (0.336–2.030)	0.677
≥ 75 years	2.488 (1.290–4.800)	**0.007**	1.643 (0.669–4.034)	0.279
Sex(female), n (%)	0.884 (0.396–1.799)	0.660		
Active smoking, n (%)	1.359 (0.659–2.802)	0.406		
Active drinking, n (%)	0.844 (0.346–2.059)	0.710		
Hypertension, n (%)	1.409 (0.690–2.878)	0.347		
Diabetes, n (%)	1.214 (0.498–2.961)	0.670		
Dyslipidemia, n (%)	0.631 (0.221–1.804)	0.390		
Left atrial dilation, n (%)	2.570 (1.256–5.256)	**0.010**	0.870 (0.347–2.179)	0.766
Heart failure, n (%)	2.986 (1.029–8.664)	**0.044**	2.052 (0.663–6.353)	0.212

*RA, right atrial; RAA, right atrial appendage; RV, right ventricle; LA, left atrial; LAA, left atrial appendage; LV, left ventricle; SUV_max_, maximum standardized uptake value; TBR, target-to-background ratio.*

*P < 0.05.*

Meanwhile, we excluded patients with relatively high ventricular uptake (52.4%, 111 cases) and divided the relatively ventricular non-uptake population into the stroke group (*n* = 15) and the non-stroke group (*n* = 86). Univariate Cox regression analyzes demonstrated that atrial fibrillation (HR = 3.533, *P* = 0.023) and high RA SUV_*max*_ (HR = 6.079, *P* = 0.001) were significantly associated with the occurrence of stroke (Supplementary Table 5). Multivariate Cox regression analysis showed that high RA SUV_*max*_ was an independent risk factor for stroke (HR = 4.260, 95% CI 1.240–14.634, *P* = 0.021).

### Addition of Atrial Fluorodeoxyglucose (FDG) Uptake to the CHA_2_DS_2_-VASc Score for Predicting Stroke

**FIGURE 4:**
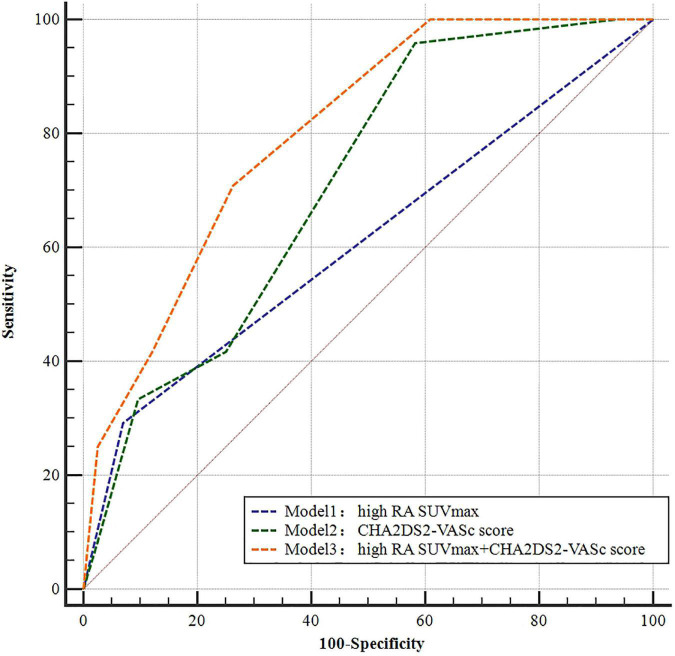
Receiver operating characteristic (ROC) curves of different models for predicting stroke.

To further assess the predictive value of atrial FDG uptake as an adjunct to the CHA_2_DS_2_-VASc score for stroke, we performed ROC curve analysis of three multivariate models (Figure 4): model 1 = high RA SUV_*max*_ (AUC = 0.636, *P* = 0.016), model 2 = the CHA_2_DS_2_-VASc score [congestive heart failure, hypertension, age ≥ 75 years (double weight), diabetes, stroke (double weight), vascular disease, age 65–74 years, sex category (female)] (AUC = 0.700, *P* < 0.001), and model 3 = high RA SUV_*max*_ + CHA_2_DS_2_-VASc score (AUC = 0.790, *P* < 0.001). The addition of high RA SUV_*max*_ to the CHA_2_DS_2_-VASc score improves the predictive value of stroke (AUC 0.790 vs. 0.700, *P* < 0.001).

### Reproducibility

Supplementary Table 6 presents the reproducibility of FDG uptake measurements. Both intra- and inter-observer comparisons showed excellent reproducibility in all of the measurements (all ICC > 0.8).

## Discussion

The main findings of the study were as follows: (1) In visual and quantitative analyzes, the atrial FDG uptake of the AF group was higher than that of the non-AF group. (2) RA FDG uptake is an independent risk factor for stroke in multivariate Cox analysis. (3) Patients with high RA SUV_*max*_ were more prone to stroke, and the median time of stroke was significantly shortened. (4) RA FDG uptake has incremental value above the CHA_2_DS_2_-VASc score in predicting stroke.

Physiological and metabolic statuses will significantly affect heart metabolism, thereby affecting the cardiac ^18^F-FDG uptake. Prolonged fasting can reduce blood glucose and insulin levels and increase free fatty acid levels, which can relatively reduce myocardial glucose utilization and improve image quality (24). Okumura et al. showed that fasting for at least 12 h can effectively inhibit the normal metabolism of cardiomyocytes, thereby highlighting the enhanced FDG uptake in inflammatory tissues (25). So, all enrolled patients were fasted before the examination (fasting time more than 12 h), which effectively inhibited the physiological uptake of the myocardium (26)

In conventional tumor ^18^F-FDG PET/CT imaging, some diseases may have influenced cardiac uptake, such as cardiac sarcoidosis, infective endocarditis, and implantable cardiac electronic device infection (27, 28). A study based on the Chinese patients showed that about 60% of patients had left ventricular myocardium uptake in oncology PET/CT, half of which were caused by myocardial ischemia (29). Therefore, in order to avoid the interference of confounding factors, we excluded cardiac diseases, other than AF, from the enrolled population. Otherwise, atrial glucose metabolism (mainly aerobic metabolism and glycolysis) is significantly lower than that of the ventricle, and the uptake of ^18^F-FDG in the atrium is almost invisible under normal circumstances (30).

Previous retrospective studies only used fasting to inhibit myocardial uptake and have shown that increased atrial FDG uptake is related to patients with AF, especially in RA (31, 32). This study also showed that whether it was visual or quantitative analysis, the atrial FDG uptake in the AF group was significantly higher than that in the non-AF group, and the differences were statistically significant. More meaningfully, Emiri et al. performed a pathological biopsy on the atria of patients with AF and found that numerous extravascular CD68-positive macrophages and CD3- or CD20-positive lymphocytes had infiltrated in the regions, demonstrating FDG uptake (21). In patients with AF, atrial pathology showed lymphatic mononuclear cell infiltration and necrosis of adjacent muscle cells, which was not present in patients with sinus rhythm (33). Several studies support a strong association between inflammation and the pathogenesis of AF (34–36). Inflammation promotes the structural remodeling and electrical remodeling of the atria, thereby shortening the atrial refractory period, causing ectopic pacing, and inducing AF (37). One hypothesis is that the increased atrial FDG uptake detected by PET may be caused by local inflammation, supported by these biopsy reports. In addition, Xie et al. explored the factors that affect atrial FDG uptake in patients with AF and found that the activity of epicardial adipose tissue (EAT) had a linear relationship with atrial FDG uptake (22). EAT is not only visceral adipose tissue but also an active endocrine organ. It can secrete a large number of inflammatory factors to act on atrial muscles, promote atrial fibrosis, lead to atrial remodeling, and provide a matrix for the occurrence and maintenance of AF (38, 39).

Meanwhile, this study also suggested that RA FDG uptake measured by ^18^F-FDG PET/CT is an independent risk factor for stroke, and the increase in RA FDG uptake is proportional to the increase in the risk of stroke. A previous study based on European populations has shown a significant correlation between atrial FDG uptake and the prevalence of stroke (20). Sinigaglia M et al. analyzed the relationship between atrial FDG uptake and stroke based on ^18^F-FDG PET/CT and found that the RA FDG uptake of stroke patients was higher than that of non-stroke patients. However, the enrolled patients were to assess cardiac inflammation or infection, and it was finally confirmed that the study population included valvular heart disease, coronary artery disease, cardiac sarcoidosis, and infective endocarditis. Those diseases and the associated confounders may have influenced atrial uptake. Moreover, this study only carried out a multivariate logistic analysis and lacked time information, which limited the ability to assess the impact of time on the occurrence of events. Also, this study neither assessed the efficacy of RA FDG uptake in predicting stroke nor compared the CHA_2_DS_2_-VASc score.

**FIGURE 5:**
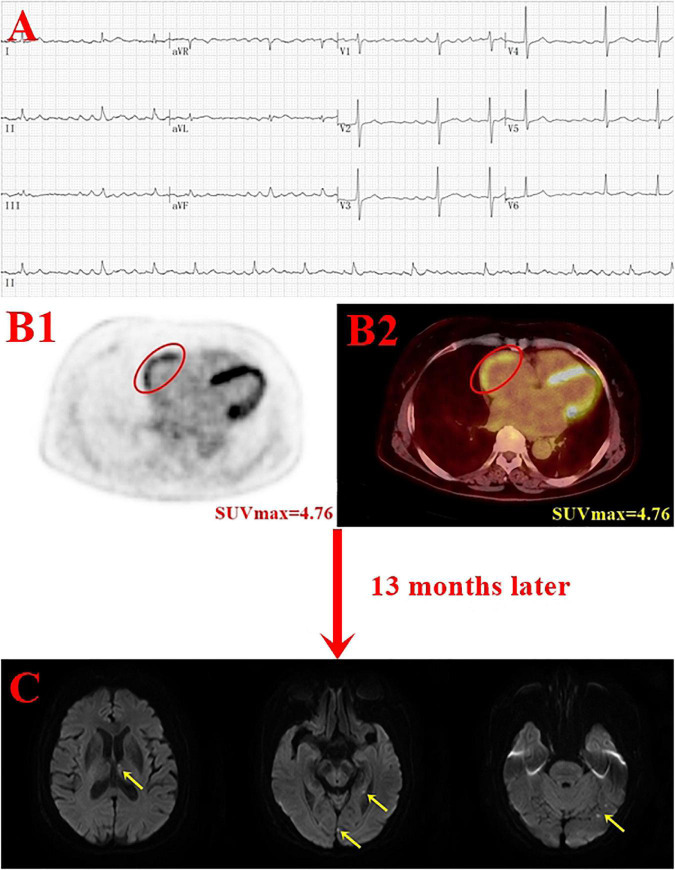
Case example. A 75-year-old woman with persistent atrial fibrillation for 8 years underwent positron emission tomography (PET)/computer tomography (CT) due to elevated CEA marker (final diagnosis of gastric cancer) and developed stroke after 13 months of the follow-up. Panel **(B)** shows the red oval circle outlines the RA FDG uptake (SUV_*max*_ = 4.76); panel C shows acute infarcts in the left thalamus, hippocampus, and occipital lobe (yellow arrow) (**A**, electrocardiogram; **B1**, PET image; **B2** PET/CT image; **C**, DWI image).

In this study, the enrolled population comprised patients who are used for tumor screening or preoperative staging, excluding cardiac and inflammatory diseases. Moreover, tumor type and treatment for tumor were not statistically different between the stroke group and the non-stroke group. Multivariate Cox regression analysis showed that high RA SUV_*max*_ (RA SUV_*max*_ ≥ 2.62) was an independent risk factor for stroke (HR = 4.227, *P* = 0.015). An elderly woman with persistentatrial fibrillation for 8 years underwent PET/CT because of elevated CEA markers. PET/CT showed RA positive uptake (SUV_*max*_ = 4.76), and stroke occurred after 13 months of follow-up (Figure 5). This study suggests that RA FDG uptake is related to the occurrence of stroke, which is consistent with previous study (20). In addition, this study found that patients with high RA SUV_*max*_ were more prone to stroke, and the median time of stroke was significantly shortened. Due to the retrospective nature of study, enrolled patients only fasted for 12 h, and half of the patients had ventricular uptake. Although the uptake of the left and right ventricles was not statistically different between the stroke group and the non-stroke group, in order to avoid the influence of the ventricle, we excluded patients with high relative ventricular uptake and performed a subgroup analysis. Univariate and multivariate Cox analyzes showed that high RA SUV_*max*_ was associated with stroke (*P* < 0.05). Consistent with the results of the whole population, it is proved that increased RA FDG uptake is an independent risk factor for stroke.

A Swedish atrial fibrillation cohort study showed that among 90,490 patients with non-valvular AF who had never received anticoagulation therapy, the C statistic of the CHA_2_DS_2_-VASc score to evaluate stroke was 0.67 (0.67–0.68) (40). In this study, the area under the CHA_2_DS_2_-VASc score curve for predicting stroke is 0.700, which is consistent with the aforementioned study. Addition of RA SUVmax to the CHA_2_DS_2_-VASc score could predict stroke more effectively, with a larger AUC 0.790 (*P* < 0.001). Our study found for the first time that adding RA FDG uptake to the CHA_2_DS_2_-VASc score could improve the incremental value for predicting stroke.

Several studies have shown that the abnormal inflammatory state may also drive the prothrombotic state in AF, which may contribute to the increased risk of thrombogenesis and, subsequently, thromboembolism (10, 41). Gregory et al. reported that CRP was positively correlated to stroke risk and related to stroke risk factors and prognosis (mortality, vascular events) (42). Yoichi et al. performed a pathological biopsy on the left atrial appendage of patients with atrial fibrillation and cardiogenic thromboembolism and confirmed that tissue factor expression induced by local inflammation is involved in the formation of thrombus in patients with AF (12). The aforementioned studies support the strong association between inflammation and pathogenesis of stroke. This provides new ideas for the mechanism research and prevention of stroke in future.

## Limitations

This study still has certain limitations. First of all, this study is a single-center study with a small sample size, which requires a large-sample prospective study for verification. Due to the limited number of studies, we could not perform subgroup analysis to explore the differences between different types of AF. Second, the enrolled population underwent ^18^F-FDG PET/CT due to tumor screening or preoperative staging, and after excluding 18 patients who died from tumor during follow-up, there were 12 (38.7%) and 72 (39.8%) tumor patients in the stroke group and the non-stroke group, respectively. Tumor may lead to stroke *via* hypercoagulability, and although the difference was not statistically significant in tumor type and treatment for tumor between the two groups of patients, the potential effects of tumor cannot be completely ruled out. In future, it is necessary to conduct relevant research on AF patients without a history of tumors. Third, due to the retrospective study, the enrolled patients only had fasted for 12 h, and half of the patients had relatively high ventricular uptake. The study showed that between the right and left ventricular uptake had no statistical difference between the stroke group and the non-stroke group, and the multivariate Cox analysis results were consistent after subgroup analysis. In future, patients need to be prepared for long-term fasting (fasting time more than 18 h), high-fat and low-carbohydrate diet, and intravenous bolus injection of heparin, so as to effectively inhibit the physiological uptake of the myocardium. Finally, this study only performed ^18^F-FDG PET/CT examinations on patients and did not perform invasive pathological biopsy on the tissues of FDG uptake in the atria, which lacked the gold standard for the diagnosis of inflammatory activity.

## Conclusion

This study found a significant correlation between atrial FDG uptake and AF, especially in RA. Meanwhile, this study found that increased RA FDG uptake is an independent risk factor for stroke, with high RA SUV_*max*_ being more prone to stroke and significantly shorter median time to stroke. Addition of RA FDG uptake to the CHA_2_DS_2_-VASc score could predict stroke more effectively and improve incremental value of traditional score. In future, it is necessary to carry out prospective studies.

## Data Availability Statement

The original contributions presented in this study are included in the article/Supplementary Material, further inquiries can be directed to the corresponding author.

## Ethics Statement

The studies involving human participants were reviewed and approved by The Third Affiliated Hospital of Soochow University. Written informed consent for participation was not required for this study in accordance with the national legislation and the institutional requirements. Written informed consent was obtained from the individual(s) for the publication of any potentially identifiable images or data included in this article.

## Author Contributions

BW wrote the draft of the manuscript. BW, YX, and PW collected and analyzed the clinical data. FZ, XS, and JW analyzed the PET/CT data. SS and YW conceived the study and interpreted the results. YW makes critical revision of the manuscript for important intellectual content. All authors contributed to the article’s revision and agreed to its submission.

## Conflict of Interest

The authors declare that the research was conducted in the absence of any commercial or financial relationships that could be construed as a potential conflict of interest.

## Publisher’s Note

All claims expressed in this article are solely those of the authors and do not necessarily represent those of their affiliated organizations, or those of the publisher, the editors and the reviewers. Any product that may be evaluated in this article, or claim that may be made by its manufacturer, is not guaranteed or endorsed by the publisher.

## References

[1] Chugh SS Havmoeller R Narayanan K Singh D Rienstra M Benjamin EJ Worldwide epidemiology of atrial fibrillation: a global burden of disease 2010 study. *Circulation.* (2014) 129:837–47. 10.1161/CIRCULATIONAHA.113.005119 24345399 PMC4151302

[2] Kornej J Börschel CS Benjamin EJ Schnabel RB. Epidemiology of atrial fibrillation in the 21st century. *Circ Res.* (2020) 127:4–20. 10.1161/CIRCRESAHA.120.316340 32716709 PMC7577553

[3] Gladstone DJ Spring M Dorian P Panzov V Thorpe KE Hall J Atrial fibrillation in patients with cryptogenic stroke. *New Engl J Med.* (2014) 370:2467–77. 10.1056/NEJMoa1311376 24963566

[4] Hindricks G Potpara T Dagres N Arbelo E Bax JJ Blomström-Lundqvist C 2020 ESC guidelines for the diagnosis and management of atrial fibrillation developed in collaboration with the European Association for Cardio-Thoracic Surgery (EACTS). *Eur Heart J.* (2021) 42:373–498. 10.1093/eurheartj/ehaa612 32860505

[5] Melgaard L Gorst-Rasmussen A Lane DA Rasmussen LH Larsen TB Lip GYH. Assessment of the CHA2DS2-VASc score in predicting ischemic stroke, thromboembolism, and death in patients with heart failure with and without atrial fibrillation. *JAMA.* (2015) 314:1030–8. 10.1001/jama.2015.10725 26318604

[6] Kornej J Apostolakis S Bollmann A Lip GYH. The emerging role of biomarkers in atrial fibrillation. *Can J Cardiol.* (2013) 29:1181–93. 10.1016/j.cjca.2013.04.016 23962731

[7] Jagadish PS Kabra R. Stroke Risk in Atrial Fibrillation: beyond the CHA2DS2-VASc Score. *Curr Cardiol Rep.* (2019) 116:1781–8. 10.1016/j.amjcard.2015.08.049 31352536

[8] Boccanelli A. Beyond CHA2DS2-VASc in atrial fibrillation: the atrium and the risk of stroke. *Eur Heart J Suppl.* (2020) 22:E30–3. 10.1093/eurheartj/suaa054 32523434 PMC7270900

[9] Wu N Chen X Cai T Wu L Xiang Y Zhang M Association of inflammatory and hemostatic markers with stroke and thromboembolic events in atrial fibrillation: a systematic review and meta-analysis. *Can J Cardiol.* (2015) 31:278–86. 10.1016/j.cjca.2014.12.002 25746020

[10] Gao SP Deng XT Ge LJ Luan H Zheng JG Chen C Is inflammation linked to thrombogenesis in atrial fibrillation? *Int J Cardiol.* (2011) 149:260–1. 10.1016/j.ijcard.2011.02.046 21402421

[11] Esenwa CC Elkind MS. Inflammatory risk factors, biomarkers and associated therapy in ischaemic stroke. *Nat Rev Neurol.* (2016) 12:594–604. 10.1038/nrneurol.2016.125 27615422

[12] Nakamura Y Nakamura K Fukushima-Kusano K Ohta K Matsubara H Hamuro T Tissue factor expression in atrial endothelia associated with nonvalvular atrial fibrillation: possible involvement in intracardiac thrombogenesis. *Thromb Res.* (2003) 111:137–42. 10.1016/S0049-3848(03)00405-5 14678810

[13] Thambidorai SK Parakh K Martin DO Shah TK Wazni O Jasper SE Relation of C-reactive protein correlates with risk of thromboembolism in patients with atrial fibrillation. *Am J Cardiol.* (2004) 94:805–7. 10.1016/j.amjcard.2004.06.011 15374796

[14] Roldan V. Interleukin-6, endothelial activation and thrombogenesis in chronic atrial fibrillation. *Eur Heart J.* (2003) 24:1373–80. 10.1016/S0195-668X(03)00239-2 12871695

[15] Hu G Liu D Tong H Huang W Hu Y Huang Y. Lipoprotein-associated phospholipase A2 activity and mass as independent risk factor of stroke: a meta-analysis. *Biomed Res Int.* (2019) 2019:1–11. 10.1155/2019/8642784 31236414 PMC6545803

[16] Jamar F Buscombe J Chiti A Christian PE Delbeke D Donohoe KJ EANM/SNMMI guideline for 18F-FDG use in inflammation and infection. *J Nucl Med.* (2013) 54:647–58. 10.2967/jnumed.112.112524 23359660

[17] Li Y Wang Q Wang X Li X Wu H Wang Q Expert consensus on clinical application of FDG PET/CT in infection and inflammation. *Ann Nucl Med.* (2020) 34:369–76. 10.1007/s12149-020-01449-8 32086761

[18] Vaidyanathan S Patel CN Scarsbrook AF Chowdhury FU. FDG PET/CT in infection and inflammation—current and emerging clinical applications. *Clin Radiol.* (2015) 70:787–800. 10.1016/j.crad.2015.03.010 25917543

[19] Irmler IM Opfermann T Gebhardt P Gajda M Bräuer R Saluz HP In vivo molecular imaging of experimental joint inflammation by combined 18F-FDG positron emission tomography and computed tomography. *Arthritis Res Ther.* (2010) 12:R203. 10.1186/ar3176 21047399 PMC3046507

[20] Sinigaglia M Mahida B Piekarski E Chequer R Mikail N Benali K FDG atrial uptake is associated with an increased prevalence of stroke in patients with atrial fibrillation. *Eur J Nucl Med Mol I.* (2019) 46:1268–75. 10.1007/s00259-019-4274-6 30680588

[21] Watanabe E Miyagawa M Uetani T Kinoshita M Kitazawa R Kurata M Positron emission tomography/computed tomography detection of increased 18F-fluorodeoxyglucose uptake in the cardiac atria of patients with atrial fibrillation. *Int J Cardiol.* (2019) 283:171–7. 10.1016/j.ijcard.2018.10.106 30420144

[22] Xie B Chen B Wu J Liu X Yang M. Factors relevant to atrial 18F-fluorodeoxyglucose uptake in atrial fibrillation. *J Nucl Cardiol.* (2020) 27:1501–12. 10.1007/s12350-018-1387-4 30088193 PMC7599132

[23] Lang RM Bierig M Devereux RB Flachskampf FA Foster E Pellikka PA Recommendations for chamber quantification: a report from the American society of echocardiography’s guidelines and standards committee and the chamber quantification writing group, developed in conjunction with the European association of echocardiography, a branch of the European society of cardiology. *J Am Soc Echocardiogr.* (2005) 18:1440–63. 10.1016/j.echo.2005.10.005 16376782

[24] Cheng VY Slomka PJ Ahlen M Thomson LEJ Waxman AD Berman DS. Impact of carbohydrate restriction with and without fatty acid loading on myocardial 18F-FDG uptake during PET: a randomized controlled trial. *J Nucl Cardiol.* (2010) 17:286–91. 10.1007/s12350-009-9179-5 20013165 PMC2842563

[25] Okumura W Iwasaki T Toyama T Iso T Arai M Oriuchi N Usefulness of fasting 18F-FDG PET in identification of cardiac sarcoidosis. *J Nucl Med.* (2004) 45:1989–98. 15585472

[26] Morooka M Moroi M Uno K Ito K Wu J Nakagawa T Long fasting is effective in inhibiting physiological myocardial 18F-FDG uptake and for evaluating active lesions of cardiac sarcoidosis. *Ejnmmi Res.* (2014) 4:1–11. 10.1186/2191-219X-4-1 24382020 PMC3880002

[27] Manabe O Koyanagawa K Hirata K Oyama-Manabe N Ohira H Aikawa T Prognostic value of 18F-FDG PET using texture analysis in cardiac sarcoidosis. *JACC Cardiovasc Imaging.* (2020) 13:1096–7. 10.1016/j.jcmg.2019.11.021 31954654

[28] Granados U Fuster D Pericas JM Llopis JL Ninot S Quintana E Diagnostic accuracy of 18F-FDG PET/CT in infective endocarditis and implantable cardiac electronic device infection: a cross-sectional study. *J Nucl Med.* (2016) 57:1726–32. 10.2967/jnumed.116.173690 27261514

[29] Wu JY Wang L Yang MF. Incidence and etiological analysis of abnormal cardiac uptake in patients underwent oncologic PET/CT imaging. *Chin J Cardiol.* (2020) 48:936–41. 10.3760/cma.j.cn112148-20191209-00742 33210865

[30] Bass A Stejskalova M Ostadal B Samanek M. Differences between atrial and ventricular energy-supplying enzymes in five mammalian species. *Physiol Res.* (1993) 42:1–06. 8329368

[31] Fujii H Ide M Yasuda S Takahashi W Shohtsu A Kubo A. Increased FDG uptake in the wall of the right atrium in people who participated in a cancer screening program with whole-body PET. *Ann Nucl Med.* (1999) 13:55–9. 10.1007/BF03165430 10202949

[32] Lobert P Brown RKJ Dvorak RA Corbett JR Kazerooni EA Wong KK. Spectrum of physiological and pathological cardiac and pericardial uptake of FDG in oncology PET-CT. *Clin Radiol.* (2013) 68:e59–71. 10.1016/j.crad.2012.09.007 23177651

[33] Frustaci A Chimenti C Bellocci F Morgante E Russo MA Maseri A. Histological substrate of atrial biopsies in patients with lone atrial fibrillation. *Circulation.* (1997) 96:1180–4. 10.1161/01.cir.96.4.1180 9286947

[34] Marott SCW Nordestgaard BG Zacho J Friberg J Jensen GB Tybjærg-Hansen A Does elevated C-reactive protein increase atrial fibrillation risk? *J Am Coll Cardiol.* (2010) 56:789–95. 10.1016/j.jacc.2010.02.066 20797493

[35] Guo Y Lip GYH Apostolakis S. Inflammation in atrial fibrillation. *J Am Coll Cardiol.* (2012) 60:2263–70. 10.1016/j.jacc.2012.04.063 23194937

[36] Van Wagoner DR Chung MK. Inflammation, inflammasome activation, and atrial fibrillation. *Circulation.* (2018) 138:2243–6. 10.1161/CIRCULATIONAHA.118.036143 30571523 PMC6334772

[37] Hu Y Chen Y Lin Y Chen S. Inflammation and the pathogenesis of atrial fibrillation. *Nat Rev Cardiol.* (2015) 12:230–43. 10.1038/nrcardio.2015.2 25622848

[38] Russo R Di Iorio B Di Lullo L Russo D. Epicardial adipose tissue: new parameter for cardiovascular risk assessment in high risk populations. *J Nephrol.* (2018) 31:847–53. 10.1007/s40620-018-0491-5 29704210

[39] Pang C Gao Z Yin J Zhang J Jia W Ye J. Macrophage infiltration into adipose tissue may promote angiogenesis for adipose tissue remodeling in obesity. *Am J Physiol Endoc M.* (2008) 295:E313–22. 10.1152/ajpendo.90296.2008 18492768 PMC2519760

[40] Friberg L Rosenqvist M Lip GY. Evaluation of risk stratification schemes for ischaemic stroke and bleeding in 182 678 patients with atrial fibrillation: the Swedish Atrial Fibrillation cohort study. *Eur Heart J.* (2012) 33:1500–10. 10.1093/eurheartj/ehr488 22246443

[41] Welsh P Lowe GDO Chalmers J Campbell DJ Rumley A Neal BC Associations of proinflammatory cytokines with the risk of recurrent stroke. *Stroke.* (2008) 39:2226–30. 10.1161/STROKEAHA.107.504498 18566306

[42] Lip GYH Patel JV Hughes E Hart RG. High-sensitivity C-reactive protein and soluble CD40 ligand as indices of inflammation and platelet activation in 880 patients with nonvalvular atrial fibrillation. *Stroke.* (2007) 38:1229–37. 10.1161/01.STR.0000260090.90508.3e 17332453

