# Supplementary material for: Right Atrial Fluorodeoxyglucose Uptake Is a Risk Factor for Stroke and Improves Prediction of Stroke Above the CHA2DS2-VASc Score in Patients With Atrial Fibrillation
Source: Front Cardiovasc Med. 2022 Jul 8;9:862000. doi: 10.3389/fcvm.2022.862000 (PMC9304590; doi:10.3389/fcvm.2022.862000)
Supplement: Supplementary file 1 [file Data_Sheet_1.docx]

Supplementary Material

**Supplementary Table 1.** ROC analysis and optimal cutoff value of cardiac uptake parameters

|  | Optimal cutoff value | AUC | Sensitivity | Specificity |
| --- | --- | --- | --- | --- |
| RA SUV_max_ | 2.62 | 0.759 | 83.9 | 66.3 |
| RAA SUV_max_ | 2.06 | 0.674 | 90.3 | 37.0 |
| RV SUV_max_ | 2.67 | 0.452 | 35.5 | 71.8 |
| LA SUV_max_ | 2.60 | 0.524 | 35.5 | 72.4 |
| LAA SUV_max_ | 2.18 | 0.636 | 80.6 | 49.7 |
| LV SUV_max_ | 6.37 | 0.507 | 51.6 | 60.8 |

RA, right atrial; RAA, right atrial appendage; RV, right ventricle; LA, left

atrial; LAA, left atrial appendage; LV, left ventricle; SUV_max_, maximum

standardized uptake value

**Supplementary Table 2.** Comparison of in tumor and medication in AF and non-AF group

|  | Total population  （N=230） | AF group  （N=115） | Non-AF group（N=115） | *P* value |
| --- | --- | --- | --- | --- |
| Tumor type, n (%) |  |  |  |  |
| Head and neck | 17（7.4） | 8（7.0） | 9（7.8） | 0.801 |
| Breast | 5（2.2） | 3（2.6） | 2（1.7） | 0.651 |
| Digestive system | 53（23.0） | 24（20.9） | 29（25.2） | 0.434 |
| Urinary system | 11（4.8） | 5（4.3） | 6（5.2） | 0.757 |
| Reproductive system | 7（3.0） | 3（2.6） | 4（3.5） | 0.701 |
| Lymphohematopoietic system | 9（3.9） | 5（4.3） | 4（3.5） | 0.734 |
| Treatment for tumor, n (%) |  |  |  |  |
| Surgery | 57（24.8） | 26（22.6） | 31（27.0） | 0.445 |
| Chemotherapy | 43（18.7） | 19（16.5） | 24（20.9） | 0.398 |
| Radiation therapy | 19（8.3） | 11（9.6） | 8（7.0） | 0.472 |
| Medication, n (%) |  |  |  |  |
| Antiplatelet | 15（6.5） | 9（7.8） | 6（5.2） | 0.423 |
| Anticoagulant | 33（14.3） | 19（16.5） | 14（12.2） | 0.347 |
| Statin | 48（20.9） | 26（22.6） | 22（19.1） | 0.516 |
| ACEI/ARB | 55（23.9） | 27（23.5） | 28（24.3） | 0.877 |
| Βeta-antagonist | 52（22.6） | 30（26.1） | 22（19.1） | 0.207 |
| Calcium antagonist | 50（21.7） | 26（22.6） | 24（20.9） | 0.749 |
| Other antihypertensive | 24（10.4） | 14（12.2） | 10（8.7） | 0.388 |

AF, atrial fibrillation; ACEI, angiotensin converting enzyme inhibitors; ARB, angiotensin receptor blocker

**Supplement table 3．**Comparison of atrial ^18^F-FDG uptake in stroke group and non-stroke group

|  | Total population  （N=212） | Stroke group  （N=31） | Non-stroke group  （N=181） | *P* value |
| --- | --- | --- | --- | --- |
| Visual analysis |  |  |  |  |
| RA positive uptake | 66（31.1） | 20（64.5） | 46（25.4） | **0.000** |
| RV positive uptake | 8（3.8） | 1（3.2） | 7（3.9） | 1.000 |
| LA positive uptake | 23（10.9） | 5（16.1） | 18（9.9） | 0.477 |
| LV positive uptake | 111（52.4） | 16（51.6） | 95（52.5） | 1.000 |
| Quantitative analysis |  |  |  |  |
| RA SUV_max_ | 2.50（1.88-3.58） | 3.60（2.80-5.10） | 2.30（1.80-3.30） | **0.000** |
| RAA SUV_max_ | 2.33（1.96-3.03） | 2.84（2.21-4.30） | 2.30（1.90-2.90） | **0.005** |
| RV SUV_max_ | 2.47（2.11-2.77） | 2.40（1.90-2.88） | 2.48（2.19-2.77） | 0.397 |
| LA SUV_max_ | 2.26（1.80-2.75） | 2.35（1.80-3.25） | 2.26（1.80-2.70） | 0.676 |
| LAA SUV_max_ | 2.25（1.90-2.76） | 2.55（2.20-3.12） | 2.20（1.89-2.70） | **0.007** |
| LV SUV_max_ | 4.99（2.83-10.33） | 6.44（3.05-11.25） | 4.99（2.83-10.09） | 0.900 |
| TBR RA | 1.28（0.75-1.91） | 1.50（1.15-2.41） | 1.22（0.75-1.78） | **0.019** |
| TBR RAA | 1.16（0.83-1.76） | 1.42（1.01-2.02） | 1.10（0.80-1.67） | **0.025** |
| TBR RV | 1.17（0.96-1.40） | 1.10（0.98-1.34） | 1.21（0.96-1.41） | 0.330 |
| TBR LA | 1.10（0.80-1.50） | 1.08（0.85-1.55） | 1.10（0.79-1.50） | 0.748 |
| TBR LAA | 1.05（0.80-1.56） | 1.17（0.87-1.56） | 1.05（0.77-1.56） | 0.263 |
| TBR LV | 2.26（1.25-5.13） | 3.18（1.45-4.57） | 2.18（1.25-5.14） | 0.877 |

AF, atrial fibrillation; RA, right atrial; RV, right ventricle; LA, left atrial; LV, left ventricle; RAA, right atrial appendage; LAA, left atrial appendage; SUV_max_, maximum standardized uptake value; TBR, target-to-background ratio

**Supplementary Table 4.** Comparison of in tumor and medication in in stroke group and non-stroke group

|  | Stroke  (N=31) | Non-stroke  (N=181) | *P* value |
| --- | --- | --- | --- |
| Tumor type, n (%) |  |  |  |
| Head and neck | 2（6.5） | 15（8.3） | 1.000 |
| Breast | 1（3.2） | 3（1.7） | 1.000 |
| Digestive system | 7（22.6） | 35（19.3） | 0.675 |
| Urinary system | 0（0） | 8（4.4） | 0.233 |
| Reproductive system | 1（3.2） | 5（2.8） | 1.000 |
| Lymphohematopoietic system | 1（3.2） | 6（3.3） | 1.000 |
| Treatment for tumor, n (%) |  |  |  |
| Surgery | 9（29.0） | 42（23.2） | 0.483 |
| Chemotherapy | 3（9.7） | 35（19.3） | 0.297 |
| Radiation therapy | 2（6.5） | 17（9.4） | 0.850 |
| Medication, n (%) |  |  |  |
| Antiplatelet | 3（9.7） | 12（6.6） | 0.816 |
| Anticoagulant | 5（16.1） | 27（14.9） | 0.862 |
| Statin | 8（25.8） | 25（13.8） | 0.089 |
| ACEI/ARB | 11（35.8） | 41（22.7） | 0.125 |
| Βeta-antagonist | 10（32.3） | 42（23.2） | 0.279 |
| Calcium antagonist | 4（12.9） | 44（24.3） | 0.242 |
| Other antihypertensive | 2（6.5） | 21（11.6） | 0.394 |

ACEI, angiotensin converting enzyme inhibitors; ARB, angiotensin receptor blocker

**Supplement table 5．**Univariate and multivariate Cox analysis of prognostic risk factors for stroke in the relatively ventricular non-uptake population

|  | Univariate analysis | | Multivariate analysis | |
| --- | --- | --- | --- | --- |
|  | HR (95% CI) | *P* value | HR (95% CI) | *P* value |
| Atrial fibrillation, n (%) | 3.533（1.190-10.488） | **0.023** | 1.921（0.544-6.789） | 0.311 |
| Tumor, n (%) | 1.141（0.413-3.150） | 0.799 |  |  |
| FDG uptake |  |  |  |  |
| High RA SUV_max_ | 6.079（2.100-17.593） | **0.001** | 4.260（1.240-14.634） | **0.021** |
| High RAA SUV_max_ | 2.801（0.787-9.973） | 0.112 |  |  |
| High LA SUV_max_ | 1.241（0.346-4.457） | 0.740 |  |  |
| High LAA SUV_max_ | 2.097（0.750-5.868） | 0.158 |  |  |
| Stroke risk factors |  |  |  |  |
| Age, n (%) |  |  |  |  |
| 74＞Age ≥ 65 years | 0.405（0.234-1.798） | 0.405 |  |  |
| ≥ 75 years | 0.921（0.230-3.688） | 0.907 |  |  |
| Sex (female), n (%) | 0.618（0.211-1.810） | 0.380 |  |  |
| Active smoking, n (%) | 1.802（0.639-5.081） | 0.265 |  |  |
| Active drinking, n (%) | 0.567（0.128-2.512） | 0.455 |  |  |
| Hypertension, n (%) | 1.375（0.489-3.867） | 0.546 |  |  |
| Diabetes, n (%) | 1.799（0.608-5.321） | 0.288 |  |  |
| Dyslipidemia, n (%) | 0.529（0.069-4.047） | 0.540 |  |  |
| Left atrial dilation, n (%) | 2.067（0.746-5.732） | 0.163 |  |  |
| Heart failure, n (%) | 2.127（0.470-9.620） | 0.327 |  |  |

RA, right atrial; RAA, right atrial appendage; RV, right ventricle; LA, left atrial; LAA, left atrial appendage; LV, left ventricle; SUV_max_, maximum standardized uptake value; TBR, target-to-background ratio

**Supplementary Table 6.** Intra- and inter-observer reproducibility

|  | Intra-observer | | Inter-observer | |
| --- | --- | --- | --- | --- |
|  | ICC (95% CI) | P value | ICC (95% CI) | P value |
| RA SUV_max_ | 0.997(0.995~0.999) | 0.000 | 0.994(0.989~0.997) | 0.000 |
| RAA SUV_max_ | 0.996(0.992~0.998) | 0.000 | 0.991(0.981~0.995) | 0.000 |
| RV SUV_max_ | 0.986(0.975~0.992) | 0.000 | 0.983(0.972~0.989) | 0.000 |
| LA SUV_max_ | 0.989(0.979~0.994) | 0.000 | 0.988(0.977~0.993) | 0.000 |
| LAA SUV_max_ | 0.987(0.975~0.993) | 0.000 | 0.983(0.971~0.991) | 0.000 |
| LV SUV_max_ | 0.981(0.970~0.989) | 0.000 | 0.978(0.968~0.984) | 0.000 |

ICC, intraclass correlation coefficient; RA, right atrial; RAA, right atrial appendage; RV, right ventricle; LA, left atrial; LAA, left atrial appendage; LV, left ventricle; SUV_max_, maximum standardized uptake value
